# Abdominal adiposity and obstructive airway disease: testing insulin resistance and sleep disordered breathing mechanisms

**DOI:** 10.1186/1471-2466-12-31

**Published:** 2012-06-28

**Authors:** Matthew T Haren, Gary Misan, Tracey-Jayne Paterson, Richard E Ruffin, Janet F Grant, Jonathan D Buckley, Peter RC Howe, Jonathan Newbury, Anne W Taylor, Robyn A McDermott

**Affiliations:** 1 Sansom Institute for Health Research, Division of Health Sciences, University of South Australia, Adelaide, SA, Australia; 2 Spencer Gulf Rural Health School (SGRHS), University of South Australia and The University of Adelaide, Whyalla Norrie, SA, Australia; 3 Centre for Rural Health and Community Development (CRHaCD), University of South Australia, Whyalla Norrie, SA, Australia; 4 Discipline of Medicine, The University of Adelaide, Adelaide, SA, Australia; 5 Population Research and Outcomes Studies, Discipline of Medicine, The University of Adelaide, Adelaide, SA, Australia; 6 Nutrition Physiology Research Centre, University of South Australia, Adelaide, SA, Australia

**Keywords:** Airway obstruction, Forced Expiratory Volume, Forced Vital Capacity, Asthma, Abdominal adiposity, Sleep disordered breathing

## Abstract

**Background:**

This study examined associations of abdominal adiposity with lung function, asthma symptoms and current doctor-diagnosed asthma and mediation by insulin resistance (IR) and sleep disordered breathing (SDB).

**Methods:**

A random sample of 2500 households was drawn from the community of Whyalla, South Australia (The Whyalla Intergenerational Study of Health, WISH February 2008 - July 2009). Seven-hundred twenty-two randomly selected adults (≥18 years) completed clinical protocols (32.2% response rate). Lung function was measured by spirometry. Post-bronchodilator FEV_1_/FVC was used to measure airway obstruction and reversibility of FEV_1_ was calculated. Current asthma was defined by self-reported doctor-diagnosis and evidence of currently active asthma. Symptom scores for asthma (CASS) and SDB were calculated. Intra-abdominal fat (IAF) was estimated using dual-energy x-ray absorptiometry (DXA). IR was calculated from fasting glucose and insulin concentrations.

**Results:**

The prevalence of current doctor-diagnosed asthma was 19.9% (95% CI 16.7 – 23.5%). The ratio of observed to expected cases given the age and sex distribution of the population was 2.4 (95%CI 2.1, 2.9). IAF was not associated with current doctor-diagnosed asthma, FEV_1_/FVC or FEV_1_ reversibility in men or women but was positively associated with CASS independent of IR and SDB in women. A 1% increase in IAF was associated with decreases of 12 mL and 20 mL in FEV_1_ and FVC respectively in men, and 4 mL and 7 mL respectively in women. SDB mediated 12% and 26% of these associations respectively in men but had minimal effects in women.

**Conclusions:**

In this population with an excess of doctor-diagnosed asthma, IAF was not a major factor in airway obstruction or doctor-diagnosed asthma, although women with higher IAF perceived more severe asthma symptoms which did not correlate with lower FEV_1_. Higher IAF was significantly associated with lower FEV_1_ and FVC and in men SDB mechanisms may contribute up to one quarter of this association.

## Background

Obesity, as defined by body mass index (BMI) has been shown to be associated with a 2.5-fold increase in the risk of current doctor-diagnosed asthma in women but not in men [1] and the association appears to be specific to non-atopic asthma [2,3]. Raviv et al. [4] showed that obesity was associated with asthma symptoms only in the least obstructed tertile, as measured by the ratio of FEV_1_ (forced expired volume in one second) to FVC (forced vital capacity). Together, these findings suggest that obesity may produce a mild obstructive airway phenotype distinct from that mediated by type 2 T-helper (Th2) cell inflammatory pathways [5]. Moreover, in one of the first studies to explore the associations between spirometric lung function and adiposity using DXA-measured fat mass, Sutherland and colleagues demonstrated correlations of total, trunk and abdominal fat masses with FVC in men and women and with FEV_1_ in men only, using a small convenience sample. This study was unable to adjust for potential confounders or investigate potential mechanisms and thus could not provide accurate estimates of the direct and indirect associations between adiposity and lung function and did not include a measurement of airway obstruction [6].

Proposed mechanisms for an obesity-induced obstructive airway phenotype include, amongst others, insulin resistance (IR) [7,8] and sleep disordered breathing (SDB) [5]. The mechanisms by which SDB could increase asthma severity are not known, but potentially involve increased vagal tone, leading to bronchoconstriction; upper airway inflammation, leading lower airway inflammation; and perturbations in central control of bronchomotor tone [5]. The authors have previously reported strong positive associations between the frequency of SDB symptoms and expression of fat mass/hyperinsulinaemia, glycaemia and lipid/lean mass phenotypes in non-diabetics in the population under study which has a prevalence of abdominal obesity of 50% [9]. Thus, this population appears suited to examine this hypothesis.

### Research context

The industrial, outer regional city of Whyalla in South Australia has a complex mix of social and physical environmental exposures which may relate to excess asthma risk. Public perceptions about the causes of respiratory ill-health in this community may be dominated by the tangible evidence of the once visible ‘red dust’ from the process of pelletising iron ore for Steelmaking and uncovered exporting of iron ore in the city [10]. An ecological study, using administrative data from 1977 to 2005 suggested that asthma, chronic lung disease, and lung cancer were in excess in Whyalla, but that the prevalence of smoking was similar in Whyalla when compared with other ‘dusty’ regional South Australian cities [11]. This suggests that local environmental or population factors might be contributing to the excess of respiratory disorders in Whyalla.

This study aimed to test the hypotheses that abdominal adiposity, which has been shown to be at high prevalence in this community, is associated with poorer lung function, increased severity of asthma symptoms and a higher prevalence of current doctor-diagnosed asthma through mechanisms involving IR or SDB.

## Methods

### Sampling and recruitment

The Whyalla Intergenerational Study of Health (WISH) cohort was established between February 2008 and July 2009. Whyalla households (n = 2500) were randomly selected from the residential housing database of the State Planning Authority. The strength of this sampling frame was the completeness, however approach and recruitment was a complex multi-stage process due to limited contact information in the databases (residential address only). This novel sampling frame was used instead of telephone listings (Electronic White Pages® (EWPs)) due to a high proportion of households without landline telephone connections, which is more common in rural than metropolitan South Australia [12]. Invitations to participate, addressed to “The Householder”, were mailed to randomly selected households, co-ordinated with a community-wide media campaign informed by a Community Advisory Group. Householders were invited to register online or by telephone, providing their telephone number and basic demographic information. One-hundred seventy-eight (7%) households responded to this approach. The second stage successfully matched 1183 of the randomly selected household addresses in the sample to names and telephone numbers in the EWPs. Thirdly, remaining unmatched randomly selected households were approached by door-knocking; a minimum of two attempts to contact were made and calling cards were left.

Once telephone numbers were obtained, Computer Assisted Telephone Interviewing (CATI) technology was used to recruit the adult (≥18 years) in each household who last had their birthday [13], and to collect demographic, health and risk factor data and schedule clinical assessments. Interviews were conducted by trained interviewers under established protocols [13,14]. Participants provided written informed consent at the time of clinical assessment. Protocols and procedures were approved by the Human Research Ethics Committee of The University of South Australia and the Aboriginal Health Research Ethics Committee of South Australia.

### Major outcome measures

Spirometry, including bronchodilator reversibility was performed in accordance with American Thoracic Society guidelines [15] and manufacturer’s recommendations using portable ultrasonic sensor spirometers (ndd EasyOne™) interfaced with EasyWare 2008 (ndd Medizintech AG, Zurich Switzerland) on laptop computers. This device has been demonstrated to maintain good stability during everyday use in Australian general practice and does not require daily calibration [16]. Quality control was maintained by achieving a minimum of three trials with volumes within 3% and achieving the manufacturers recommended minimum system quality control grade. The primary spirometric outcome was airway obstruction measured by post-bronchodilator FEV_1_/FVC. The degree of reversibility in the FEV_1_ was calculated as: 100 x FEV_1_ (post-bronchodilator) – FEV_1,_(baseline)/ FEV_1_ (baseline). Secondary outcomes were post-bronchodilator FEV_1_; FVC; peak expiratory flow (PEF); and the average expired flow over the middle half of the FVC manoeuvre (FEF_25-75%_). These variables were also referenced to South Australian-derived prediction equations of Gore et al. [17] and expressed as the percentage of predicted value (PPV).

Self-reported ‘ever doctor-diagnosed asthma’ was defined as answering yes to either: (1) Have you ever been told by a doctor that you have any of the following conditions? (option 2 = Asthma); or (2) have you ever been told by a doctor that you have asthma? ‘Current doctor-diagnosed asthma’ was defined as ‘ever doctor-diagnosed asthma’ plus evidence of currently active asthma defined by positive responses to any of the following questions: (1) During the past 12 months, did you have any symptoms of asthma? (2) During the past 12 months, did you take asthma medication that was prescribed or given to you by a doctor? (3) Do you still have asthma? The frequency of three asthma symptoms (wheeze, shortness of breath and cough; scored as never (0), occasionally (1), most days/every day (2)) and the amount of phlegm produced during cough (none (0), a little (1), several tablespoons/day or more (2)) over the past three months [18], were summed to produce a comprehensive asthma symptom score (CASS) ranging from zero indicating no asthma symptoms to eight indicating the highly frequent presence of all four symptoms.

### Independent variables

Body composition was assessed using dual-energy x-ray absorptiometry (DXA, Lunar Prodigy, GE Medical Systems, Madison WI) with the manufacturer’s software (enCORE 2003 version 7.52). Scans were performed according to the manufacturer’s protocols with participants supine. Total body fat mass (g) was determined automatically from the manufacturer’s software. Total intra-abdominal fat (IAF) mass was estimated by defining a region of interest in the abdomen by drawing quadrilateral box with the base touching the top of the iliac crest, the upper margins touching the most inferior aspect of the ribs and the lateral borders extending to the inner border of the rib cage. IAF percentage was estimated as fat mass / (fat mass + lean mass + bone mineral content) * 100.

Fasting, morning venous blood samples were collected (approximately 40 mL) and assayed by a National Association of Testing Authorities accredited laboratory. Fasting plasma glucose concentrations were measured using a Chemistry analyser system (Olympus AU5400, Olympus Optical C Ltd, Japan) with inter-assay CVs of 2.41% at 3.41 mmol/L and 2.21% at 19.72 mmol/L. Fasting serum insulin concentrations were measured in serum collected, separated and frozen at −80°C within two hours of collection. Radioimmunoassay was performed on an Abbott Architect immunoassay analyser (Abbott Park, IL USA) with inter-assay CVs of 7.81% at 8.44 mU/L, 5.87% at 57.44 mU/L and 8.53% at 97.90 mU/L. IR was estimated from these two concentrations using the Homeostasis Model Assessment (HOMA2) Microsoft Excel calculator from the University of Oxford Diabetes Trial Unit (http://www.dtu.ox.ac.uk/homacalculator/index.php) which uses the equations of Levy and colleagues [19]. A SDB symptom score (range 0–12) was computed as the sum of symptom frequency from zero (never) to four (>4 times per week) for three symptoms: snorting or gasping; loud snoring; and breathing cessation, choking or struggling for breath during sleep [20].

### Covariates

Height was determined to within one millimetre using a wall stadiometer (Surgical and Medical Products No. 26SM, Mentone Education Supplies, Melbourne, Australia), body mass was measured to the nearest 100 g with an electronic scale (Tanita BF-681). Smoking status was defined as ‘never smoker’, ‘past smoker’ and ‘current smoker’. Gross annual household income was collected in approximate 20,000 Australian Dollar (AUD) increments and reduced to three categories of 40,000 AUD increments for analysis. The number of people residing in the household was categorised as one, two, three, four, and five or more. Medication inventory was performed during clinical assessment and participants were asked if they had taken bronchodilators or any other respiratory medication in the previous 24 hours. Doctor-diagnosis of Obstructive Sleep Apnoea Syndrome (OSAS) and management of OSAS with Continuous Positive Airway Pressure (CPAP) therapy was determined by questionnaire.

### Statistical analysis

Data analysis was performed using Stata 11 for Windows (StataCorp, College Station TX USA). For prevalence estimates, data were weighted to the age and sex distribution of the Estimated Residential Population of Whyalla in 2007 [21] and the likelihood of being recruited into the study, as per the equation: weight = hhld_adult*(bnh/lnh)*(ln/bn); where hhld_adult is the number of people aged 18 and over (adults) residing in the household; bnh is the adult population size of Whyalla by sex and age-group; bn is the total adult population of Whyalla; lnh is the adult sample size of WISH by sex and age-group; and ln is the total adult sample size of the WISH cohort. To calculate the Australian standardised prevalence ratio (SPR) for current doctor diagnosed asthma in Whyalla, the stratum-specific prevalence estimates from the national standard population data [22] were used to calculate the expected number of cases in the study population (WISH). Expected cases were summed across strata, and compared with the number of observed cases, the SPR was calculated as the ratio of observed to expected cases. The same method was used to estimate the SPR’s for current and past smoking using 2007–2008 Australian NHS data as the standard population.

**Figure 1:**
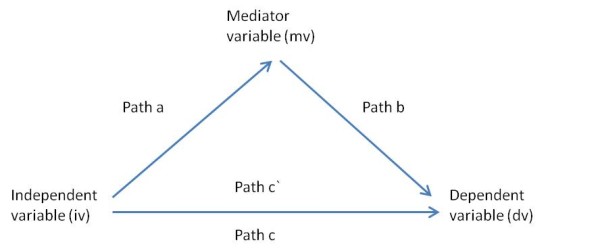
**Regression Paths in Mediation Analysis.** The mediation model of Baron & Kenny, 1986 [24]. Criteria for mediation are: (1) significant *path c* association; (2) significant *path a* association; (3) significant *path b* association with a reduction in the *path c* coefficient ( *c`*).

Ordinary least squares regression was used to model the associations of IAF with: spirometric outcomes and log-CASS. Prevalence ratios (PR) for the association of IAF with current doctor diagnosed asthma were estimated using Generalised Linear Models with Poisson distribution, log-link function and robust estimation of confidence intervals as suggested by Wolkewitz et al. 2007 [23]. Formal mediation tests were conducted in Stata 11 using -*sgmediation-,* which uses bootstrap analyses to estimate the indirect effect of the independent variable on the dependent variable through the mediator variable, overcoming the assumption of a normal sampling distribution required for use of the classical Sobel test. Bootstrap analysis involves drawing a large number of samples, in this case 5000 (with replacement), from the data set, computing the indirect effect for each sample, and then generating an average indirect effect across all samples. Regression paths and criteria for mediation followed those outlined by Baron and Kenny [24] and summarised in Figure 1.

## Results

**Figure 2:**
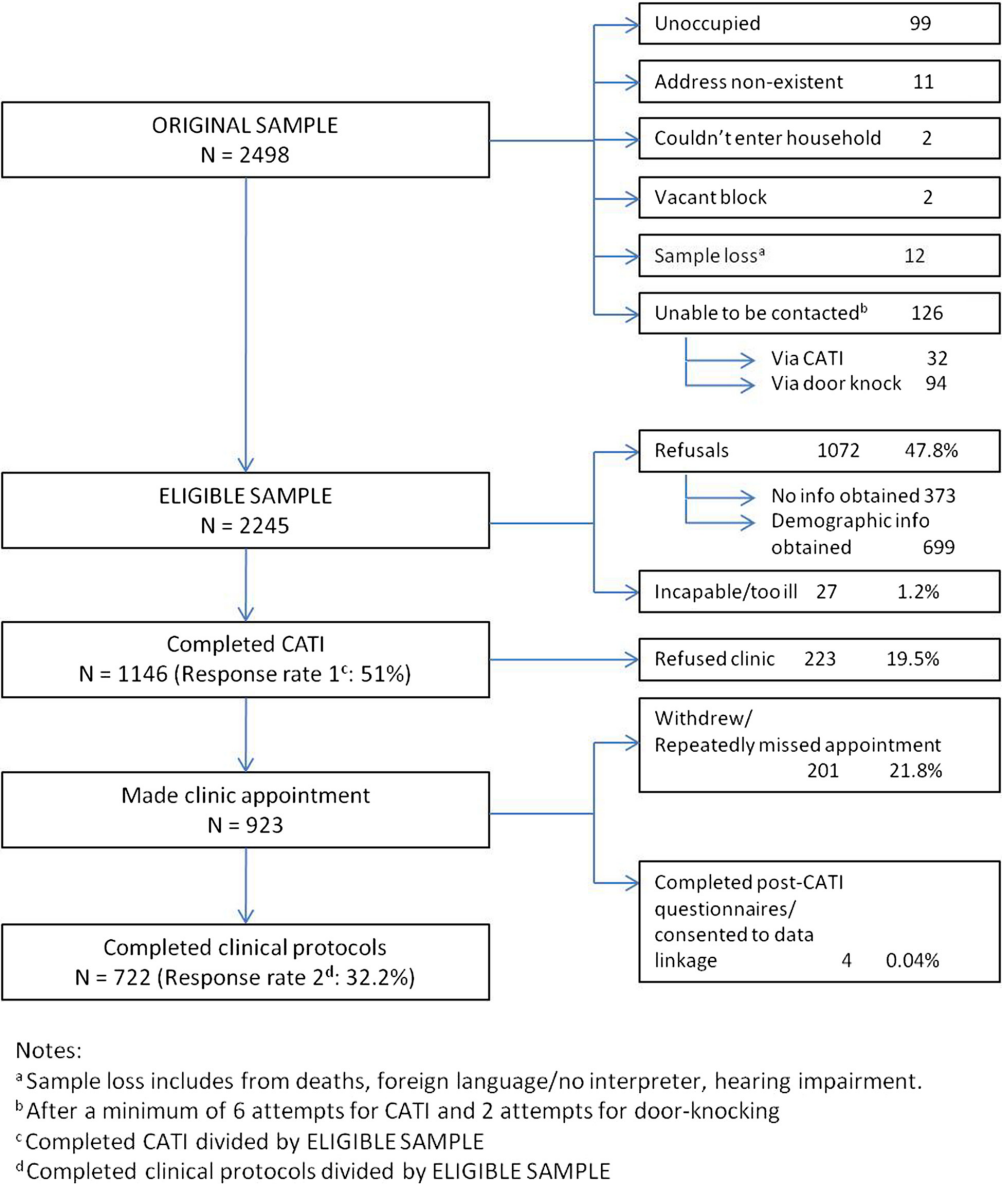
Flowchart of participation in the Whyalla Intergenerational Study of Health (WISH, 2008–2009).

Overall, 51% of eligible households participated in the CATI and 32.2% participated in the clinical study (Figure 2).

The estimated population prevalence of current doctor-diagnosed asthma was 19.9% [95% CI 16.7 – 23.5%] which was 2.4 times higher than Australian national prevalence estimates after age- and sex-standardisation [95%CI for the SPR 2.1 – 2.9]. The prevalence of both current (23% [95% CI 19.6 – 26.7%]; SPR 1.11 [95% CI 0.95 - 1.29]) and past smoking (28.6% [95% CI 25.1 – 32.4%]; SPR 0.96 [95% CI 0.83 - 1.10]) in Whyalla were similar to those of Australia overall.

Of the 726 adults who completed comprehensive questionnaires, 236 men and 348 women (n = 562, 78%) completed baseline and post-bronchodilator spirometric manoeuvres of acceptable quality. In total, 98 participants did not complete spirometry tests for various reasons: opted out (n = 29); terminated spirometry protocols early (n = 66); excluded on the basis of illness, injury or doctor’s advice (n = 3). Sixty-six participants completed spirometry, but breathing manoeuvres were of unacceptable quality to provide valid and reliable data (n = 60) or technical malfunctions were experienced (n = 6).

Post-bronchodilator FEV_1_/FVC, FEV_1_, PEF and FEF_25%-75%_ were all lower and there was a non-significant trend towards higher FEV_1_ reversibility in current doctor-diagnosed asthmatics when compared with non-asthmatics. CASS was higher in current doctor-diagnosed asthmatics when compared with non-asthmatics but it did not differ by spirometry completion or quality grade in either group (Table 1). Neither IR nor SDB symptom scores were higher in the current doctor-diagnosed asthmatics when compared with non-asthmatics, but within the doctor-diagnosed group, IR was higher in those who did not attempt or complete an acceptable spirometry test (Table 1). A complete respiratory, metabolic and socio-demographic description of the study sample by asthma diagnosis is presented in Additional file 1.

**Table 1:** Respiratory, metabolic and socio-demographic characteristics, The Whyalla Intergenerational Study of Health (WISH, 2008–2009)

		**Current Doctor-Diagnosed Asthma**				**Asthma x spirometry quality**	
		**No (n = 575)**	**Yes (n = 151)**	**F(df) or Chi2(df)**	**P**	**F(df) or Chi2(df)**	**P**
***Completion/***	No test	77	21				
***acceptability of spirometry***	Unacceptable	51	15				
	Acceptable	447	115				
***Outcomes, asthma symptoms***							
CASS, range 0-8	No test	3.62 (3.26, 3.99)	5.33 (4.64, 6.27)	F(5,711) = 19.26	<0.001	F(2,711) = 1.16	0.32
	Unacceptable	3.28 (2.83, 3.72)	5.23 (4.35, 6.11)				
	Acceptable	3.28 (3.13, 3.43)	4.56 (4.26, 4.86)				
log CASS	No test	1.16 (1.05, 1.27)	1.63 (1.41, 1.84)	F(5,711) = 14.33	<0.001	F(2,711) = 0.55	0.58
	Unacceptable	1.09 (0.95, 1.22)	1.54 (1.27, 1.82)				
	Acceptable	1.07 (1.03, 1.12)	1.42 (1.33, 1.51)				
***Outcomes, spirometry***							
***(acceptable tests only)***							
FEV1 reversibility, L		0.11 (0.09, 0.12)	0.12 (0.09, 0.14)	F(1, 538) = 0.22	0.64		
FEV1 reversibility,%		3.88 (3.27, 4.49)	5.03 (3.83, 6.24)	F(1, 538) = 2.80	0.10		
post FEV1, L		3.01 (2.93, 3.10)	2.76 (2.60, 2.92)	F(1, 538) = 7.62	<0.01		
post FVC, L		3.80 (3.70, 3.90)	3.64 (3.44, 3.83)	F(1, 523) = 2.02	0.16		
post FEV1/FVC,%		79.46 (78.73, 80.19)	76.14 (74.70, 77.57)	F(1, 523) = 16.34	<0.001		
post PEF, L/s		7.83 (7.62, 8.03)	7.23 (6.83, 7.63)	F(1, 538) = 6.80	<0.01		
post FEF25%-75%, L/s		3.06 (2.94, 3.17)	2.55 (2.32, 2.78)	F(1, 538) = 15.16	<0.001		
FEV1, ppv†		90.34 (88.79, 91.88)	84.20 (81.16, 87.24)	F(1, 538) = 12.46	<0.001		
FVC, ppv†		93.90 (92.44, 95.35)	91.70 (88.81, 94.60)	F(1, 523) = 1.76	0.19		
FEV1/FVC, ppv†		96.66 (95.81, 97.50)	91.03 (89.35, 92.70)	F(1, 523) = 34.55	<0.001		
PEF, ppv†		97.24 (95.50, 98.98)	92.44 (89.02, 95.85)	F(1, 538) = 6.03	0.02		
FEF25%-75%, ppv†		84.55 (81.90, 87.20)	71.28 (66.07, 76.49)	F(1, 538) = 19.79	<0.001		
***Independent variables***							
Intra-abdominal fat,%	No test	39.58 (37.26, 41.90)	39.44 (34.92, 43.96)	F(5,692) = 1.16	0.33	F(2,692) = 1.71	0.18
	Unacceptable	38.81 (35.92, 41.70)	33.06 (27.66, 38.47)				
	Acceptable	39.55 (38.57, 40.52)	39.91 (37.99, 41.83)				
***Mediator variables***							
HOMA2-IR	No test	1.35 (1.09, 1.66)	2.01 (1.52, 2.52)	F(5,703) = 1.51	0.19	F(2,703) = 3.18	0.04
	Unacceptable	1.39 (1.06, 1.71)	1.71 (1.09, 2.34)				
	Acceptable	1.40 (1.30, 1.59)	1.32 (1.11, 1.54)				
SDB symptom score, range 0-12	No test	1.18 (0.63, 1.73)	1.87 (0.75, 2.98)	F(5,594) = 1.74	0.12	F(2,594) = 0.07	0.93
	Unacceptable	1.54 (0.83, 2.25)	2.09 (0.79, 3.40)				
	Acceptable	0.96 (0.74, 1.18)	1.39 (0.93, 1.85)				

A description of the outcome, independent and mediator variables, and covariates by current doctor-diagnosed asthma and spirometry completion and quality, The Whyalla Intergenerational Study of Health (WISH, 2008–2009).

Determination of ‘unacceptable test’ was based on either baseline and post-bronchodilator EasyOne™ system quality control (QC) grade of D or lower, or non-reproducible trials exceeding 3% variability. The analytical sample satisfied a QC grade of C or higher and reproducible trials within 3% variability. †Percentage of Predicted Value (PPV) was based on equations of Gore et al. (1995) [17].

Obstructive sleep apnoea syndrome (OSAS) had been doctor-diagnosed in 42 participants (5.9%) and 20 of these used CPAP to manage the condition. CPAP use was associated with a lower log CASS (1.18 ± 0.56 v 1.52 ± 0.40, p = 0.04) but non-significant differences in current doctor diagnosed asthma prevalence, IAF, IR, SDB symptom score, post FEV_1_/FVC and reversibility of FEV_1_ (data not shown).

Table 2 shows the covariate-adjusted associations of IAF with current doctor-diagnosed asthma, CASS, spirometric outcomes, IR and SDB symptom frequency in men and women who completed acceptable spirometry tests. IAF was not associated with current doctor-diagnosed asthma, FEV_1_/FVC, FEV_1_ reversibility, FEF_25-75%_ or PEF and in men or women. A one percent increase in IAF was associated with a significant >1 unit increase in CASS in both men and women (0.006 and 0.009 respectively on the log scale) although the association in men was of borderline significance and was not robust to repeated bootstrap analysis. The associations of CASS with both FEV_1_/FVC and FEV_1_ were different between men and women. For every log-unit increase in CASS FEV_1_/FVC declined by 4% (p <0.001) and FEV_1_ by 295 mL in men (p = 0.007) but there were no associations in women.

**Table 2:** Associations of intra-abdominal fat percentage with lung function and asthma outcomes, and hypothesised mediators (sleep disordered breathing score and insulin resistance) in men and women, The Whyalla Intergenerational Study of Health (WISH, 2008–2009)

	**Men**			**Women**		
	**Coef. (bootstrap 95%CI)**	**P**	**R-squared**	**Coef. (bootstrap 95%CI)**	**P**	**R-squared**
Current doctor-diagnosed asthma	1.002 (0.975, 1.029)	0.913		1.004 (0.983, 1.027)	0.686	
*Obs*	*214*			*320*		
log CASS	0.006 (0.000, 0.012)	0.045	0.148	0.009 (0.002, 0.016)	0.011	0.123
*Obs*	*213*			*317*		
rev FEV1	0.045 (−0.045, 0.135)	0.328	0.077	0.005 (−0.063, 0.073)	0.892	0.026
*Obs*	*212*			*313*		
post FEV1/FVC						
L/L	0.065 (−0.028, 0.158)	0.172	0.363	0.056 (−0.002, 0.113)	0.056	0.321
PPV (Gore et al. [17])	0.090 (−0.029, 0.209)	0.138	0.229	0.066 (−0.033, 0.165)	0.194	0.129
*Obs*	*210*			*300*		
SDB	0.055 (0.017, 0.094)	0.005	0.129	0.024 (0.002, 0.047)	0.034	0.083
*Obs*	*178*			*273*		
HOMA2-IR	0.059 (0.048, 0.071)	<0.001	0.272	0.052 (0.038, 0.065)	<0.001	0.286
*Obs*	*214*			*318*		

Associations of intra-abdominal fat percentage with lung function and asthma outcomes, and hypothesised mediators (sleep disordered breathing score and insulin resistance) in men and women, The Whyalla Intergenerational Study of Health (WISH, 2008–2009). FEV_1_, forced expiratory volume in 1 second; FVC, forced vital capacity; IR, insulin resistance (HOMA2); SDB, sleep-disordered breathing. Covariates included in all models were age, current and past smoking, gross annual household income and number of household residents. For lung function models height and respiratory medication in the previous 24 hours were included as covariates.

A one percent increase in IAF was associated with a 12 ml and 20 mL decrease in FEV_1_ and FVC respectively in men and a 4 ml and 7 mL decrease respectively in women. This corresponded to reductions in the percentage of predicted value for FEV_1_ of 34% in men and 16% in women; and for FVC of 46% in men and 24% in women. IAF was positively associated with IR and SDB symptom frequency in both men and women, although the significance of the association at p <0.05 between SDB and IAF in women was not robust to repeated bootstrap analysis.

The significant associations of IAF with CASS, FEV_1_ and FVC were tested for mediation by IR and SDB symptom frequency in men and women. After adjustment for IAF, IR was not associated with any of the three outcomes in men or women (path b, data not shown) and thus did not meet the necessary criteria for mediation. In men, after adjustment for IAF, SDB frequency was significantly associated with FEV_1_ and FVC (path b) and mediated between 12% and 26% of the associations with IAF (Table 3). The small total ‘effect’(association) between IAF and CASS in men did not reach significance during mediation analysis and thus the 65% mediation of that association is of little significance. In women, associations between the SDB and IAF were weak and of borderline significance corresponding to relatively minor indirect effects.

**Table 3:** **Mediation by sleep-disordered breathing symptom frequency of associations of intra-abdominal fat with comprehensive asthma symptom score, FEV**_**1**_**and FVC expressed as Litres and ‘percentage of predicted values’ (PPV) in men and women, The Whyalla Intergenerational Study of Health (WISH, 2008–2009)**

	**Path a**	**Path b**	**Total effect (Path c)**	**Direct effect (Path c')**	**Bootstrapped**	%
***Dependent variables (dv)***	**mv regressed on IAF**	**dv regressed on mv**	**dv regressed on IAF**	**dv regressed on IAF**	**Indirect Effect**	**Mediated**
		**controlling for IAF**		**controlling for mv**		
*MEN*						
log CASS, *n = 178*	0.060 (0.025, 0.094)	0.061 (0.027, 0.095)	0.006 (−0.002, 0.013)	0.002 (−0.006, 0.010)	0.004 (0.001, 0.007)	64.7
post FEV_1_, *n = 178*						
Litres	0.060 (0.025, 0.094)	−0.047 (−0.080, -0.014)	−0.012 (−0.019, -0.005)	−0.009 (−0.017, -0.002)	−0.003 (−0.006, -0.001)	23.4
PPV *(Gore et al.*[17]*)*	0.061 (0.026, 0.096)	−1.462 (−2.370, -0.555)	−0.344 (−0.553, -0.135)	−0.255 (−0.466, -0.045)	−0.087 (−0.187, -0.026)	25.8
post FVC, *n = 176*						
Litres	0.060 (0.026, 0.095)	−0.040 (−0.079, -0.002)	−0.020 (−0.029, -0.011)	−0.018 (−0.027, -0.009)	−0.002 (−0.006, -0.000)	12.1
PPV *(Gore et al.*[17]*)*	0.061 (0.026, 0.096)	−1.271 (−2.104, -0.439)	−0.467 (−0.658, -0.275)	−0.389 (−0.583, -0.196)	−0.076 (−0.154, -0.025)	16.6
*WOMEN*						
log CASS, *n = 271*	0.024 (−0.001, 0.049)	0.054 (0.023, 0.085)	0.010 (0.003, 0.016)	0.008 (0.002, 0.015)	0.001 (0.000, 0.003)	13.7
post FEV_1_, *n = 267*						
Litres	0.021 (−0.004, 0.047)	−0.018 (−0.044, 0.009)	−0.005 (−0.010, 0.001)	−0.004 (−0.010, 0.002)	−0.000 (−0.002, 0.000)	8.5
PPV *(Gore et al.*[17]*)*	0.021 (−0.004, 0.047)	−0.821 (−1.810, 0.167)	−0.204 (−0.409, 0.001)	−0.186 (−0.392, 0.020)	−0.019 (−0.066, 0.001)	8.6
post FVC, *n = 254*						
Litres	0.022 (−0.005, 0.048)	−0.013 (−0.044, 0.019)	−0.008 (−0.014, -0.001)	−0.007 (−0.014, -0.001)	−0.000 (−0.002, 0.000)	3.7
PPV *(Gore et al.*[17]*)*	0.021 (−0.005, 0.048)	−0.587 (−1.551, 0.377)	−0.263 (−0.461, -0.064)	−0.250 (−0.449, -0.051)	−0.013 (−0.065, 0.006)	4.8

Mediation by sleep-disordered breathing symptom frequency of associations of intra-abdominal fat with comprehensive asthma symptom score, FEV_1_ and FVC expressed as volumes and ‘percentage of predicted values’ (PPV) in men and women, The Whyalla Intergenerational Study of Health (WISH, 2008–2009).

CASS, comprehensive asthma symptom score; FEV_1_, forced expiratory volume in 1 second; FVC, forced vital capacity; PPV, Percentage of Predicted Value. Criteria for mediation are: (1) significant *path c* association; (2) significant *path a* association; (3) significant *path b* association with a reduction in the *path c* coefficient ( *c`*). Covariates included in all models were age, current and past smoking, gross annual household income and number of household residents. For lung function models height and respiratory medication in the previous 24 hours were included as covariates.

## Discussion

In this population there was little evidence to support the existence of an obesity-induced obstructive airway phenotype. These findings are inconsistent with the previously documented associations between central obesity and asthma, particularly mild non-atopic asthma phenotypes [1-3,25]. This may suggest the dominance of other asthma-causing agents over obesity in this population. The only indication of a link between abdominal adiposity and asthma was the association between greater IAF and more frequent asthma symptoms, particularly in women where there was no relation to measured airway obstruction. Thus, this association may be partly explained by greater perceived exertion from daily physical tasks in more abdominally obese people rather than true airway obstruction.

The contribution of the high prevalence of abdominal obesity to the relative 2.4 case excess of current doctor-diagnosed asthma in this community would appear therefore to be low and instead be dominated by non-obesity induced phenotypes. Asthma symptoms were higher and post-bronchodilator lung function was poorer in those who reported a current doctor-diagnosis of asthma, but as previously noted asthma symptom scores were poorly correlated with measured airway obstruction in women, who were significantly more likely than men to report a current doctor-diagnosis of asthma. Anecdotally, there is high awareness of respiratory disease in this city which may contribute to greater knowledge of respiratory symptoms, higher levels of help-seeking by those experiencing respiratory symptoms (which may be biased towards women) and more widespread asthma screening and diagnosis by general practitioners. In terms of other potentially contributing population factors, the city has a greater than twenty percent enrichment of blue-collar workers when compared with South Australia and Australia overall [26], who are largely employed in the steel industry. This may confer significantly more occupational asthma on the population over and above the 15% of cases in working aged adults estimated to be either caused or aggravated by occupational exposures from previous research [27]. The degree to which occupational exposures, environmental dust exposure or other population factors such as asthma awareness (population and general practitioners) and vigilance in help-seeking contribute to the excess of, and gender difference in, current doctor-diagnosed asthma remains to be determined in this community which has implications for community interventions on asthma.

IAF was, however, associated with FEV_1_ and FVC in the Whyalla population. Based on age-related rates of decline in lung volumes from the US National Health and Nutrition Examination Survey (NHANES) [28] a five percent increase in IAF (adjusted for IR or SDB symptom frequency) was equivalent to two years of age-related decline in the FEV_1_ (60 ml) and more than three years of age-related decline in the FVC (100 ml) of men; the effects were up to three times smaller in women. These associations, including being three to four times weaker in women compared with men, were of similar magnitude to those of other studies [25,29-31], although the range of adiposity measures used make cross-study comparisons difficult.

We did not observe the inverse association between IR and expired lung volumes previously reported in cross-sectional studies of other populations [32,33]. Prospective studies have shown that reduced lung function can precede the development of IR and diabetes [34-39] possibly through microangiopathy of alveolar capillaries and pulmonary arterioles [40,41]. Thus it is possible that the absence of a cross-sectional association between lung function and IR in this study may be due to healthy-participant bias. If those with more advanced disease (reduced lung function) and IR, were less likely to participate due to poor health (or having died) than those with earlier stage reductions in lung function without IR, then the association between lung function and IR would be underestimated.

Associations between IAF and SDB symptom frequency were weaker in women than in men and had negligible mediating effects on FEV_1_, FVC or CASS. In men however, SDB symptom frequency mediated between 12% and 17% of the association of IAF with FVC and between 23% and 26% of the association with FEV_1_. This finding is inconsistent with two small studies (n ≤20) that suggested six to eight weeks of continuous positive airway pressure treatment for sleep apnoea was not effective in improve spirometric measures of lung function [42,43] but improved asthma symptoms. These studies were unlikely to have been powered to detect meaningful changes in these outcomes, but they do raise caution about interpreting causal directions from these cross-sectional findings.

There is no universally applied clinical definition of asthma and case assignment in epidemiological studies is often defined by self-reported doctor-diagnosis, which has good sensitivity (91%), specificity (97%) and reasonable positive predictive value (60%) for indicating prevalent disease [44]. The combination of measured lung function, self-reported asthma symptom frequency and doctor-diagnosed current asthma as outcomes; and the specific focus on intra-abdominal fat estimated using DXA measurements as opposed to most other studies which have relied on BMI or waist girths as measures of adiposity are particular strengths of this study. Assessment of SDB was limited to self-reported symptom frequencies which have good sensitivity (85%) but poor specificity (55%) for identifying men with obstructive sleep apnoea [20].

In terms of potential selection bias, this cohort was 10% under-represented by state-administered housing residents which, due to greater odds of current doctor-diagnosed asthma in lower-income households combined with greater odds of abdominal obesity and diabetes in households with gross annual income below 40,000 AUD in this community (unpublished observations), would theoretically result in an underestimate of the true abdominal adiposity-current doctor diagnosed asthma association. However, given the strength of the null finding it is unlikely that elimination of this potential bias would shift the association to a degree which would result in statistical or clinical significance. In Table 1, we showed that within the current doctor-diagnosed asthmatics, IR was higher in those who did not attempt or complete an acceptable spirometry test. As stated previously, if those asthmatics with more severe IR for which acceptable quality spirometric data were not available also had poorer lung function, than those without IR, then the association between lung function and IR would theoretically be underestimated. However, Table 1 also reveals evidence that CASS did not differ by spirometry completion or quality grade in either current doctor-diagnosed asthmatics or non-asthmatics which suggests non-completion of successful spirometry was not dependent on greater obstructive airway symptom severity indicating that this may in fact not be a source of bias.

## Conclusions

In this population with a high prevalence of abdominal obesity and an excess of doctor-diagnosed asthma, IAF was not a major factor in doctor-diagnosed asthma or airway obstruction although women with higher IAF perceived more severe asthma symptoms which did not correlate with lower FEV_1_. Higher IAF was significantly associated with lower FEV_1_ and FVC and in men SDB mechanisms may contribute up to one quarter of this association.

## Competing interests

The authors declare no competing interests.

## Authors’ contributions

All authors with the exception of TJP participated in the design of the study. All authors contributed to the interpretation of results and reviewing the manuscript for important intellectual content. Additionally, MTH and TJP participated in data collection. MTH performed the statistical analysis and drafted the manuscript. All authors read and approved the final manuscript.

## Pre-publication history

The pre-publication history for this paper can be accessed here:

http://www.biomedcentral.com/1471-2466/12/31/prepub

## Supplementary Material

Additional file 1 **Table S1.** Respiratory, metabolic and socio-demographic characteristics, The Whyalla Intergenerational Study of Health (WISH, 2008-2009). Click here for file
