# Supplementary material for: Abdominal adiposity and obstructive airway disease: testing insulin resistance and sleep disordered breathing mechanisms
Source: BMC Pulm Med. 2012 Jun 28;12:31. doi: 10.1186/1471-2466-12-31 (PMC3544645; doi:10.1186/1471-2466-12-31)
Supplement: Additional file 1 — Table S1. Respiratory, metabolic and socio-demographic characteristics, The Whyalla Intergenerational Study of Health (WISH, 2008-2009). [file 1471-2466-12-31-S1.doc]

**Supplementary Table 1. Respiratory, metabolic and socio-demographic characteristics, The Whyalla Intergenerational Study of Health (WISH, 2008-2009).**

|  |  | Current Doctor-Diagnosed Asthma | |  | | Asthma x spirometry quality | |
| --- | --- | --- | --- | --- | --- | --- | --- |
|  |  | No (n=575) | Yes (n=151) | F(df) or Chi2(df) | P | F(df) or Chi2(df) | P |
| ***Completion/*** | No test | 77 | 21 |  |  |  |  |
| ***acceptability of spirometry*** | Unacceptable | 51 | 15 |  |  |  |  |
|  | Acceptable | 447 | 115 |  |  |  |  |
| ***Outcomes, asthma symptoms*** |  |  |  |  |  |  |  |
| CASS, range 0-8 | No test | 3.62 (3.26, 3.99) | 5.33 (4.64, 6.27) | F(5,711) = 19.26 | <0.001 | F(2,711) = 1.16 | 0.32 |
|  | Unacceptable | 3.28 (2.83, 3.72) | 5.23 (4.35, 6.11) |  |  |  |  |
|  | Acceptable | 3.28 (3.13, 3.43) | 4.56 (4.26, 4.86) |  |  |  |  |
|  |  |  |  |  |  |  |  |
| log CASS | No test | 1.16 (1.05, 1.27) | 1.63 (1.41, 1.84) | F(5,711) = 14.33 | <0.001 | F(2,711) = 0.55 | 0.58 |
|  | Unacceptable | 1.09 (0.95, 1.22) | 1.54 (1.27, 1.82) |  |  |  |  |
|  | Acceptable | 1.07 (1.03, 1.12) | 1.42 (1.33, 1.51) |  |  |  |  |
| ***Outcomes, spirometry*** |  |  |  |  |  |  |  |
| ***(acceptable tests only)*** |  |  |  |  |  |  |  |
| FEV1 reversibility, L |  | 0.11 (0.09, 0.12) | 0.12 (0.09, 0.14) | F(1, 538) = 0.22 | 0.64 |  |  |
| FEV1 reversibility, % |  | 3.88 (3.27, 4.49) | 5.03 (3.83, 6.24) | F(1, 538) = 2.80 | 0.10 |  |  |
| post FEV1, L |  | 3.01 (2.93, 3.10) | 2.76 (2.60, 2.92) | F(1, 538) = 7.62 | <0.01 |  |  |
| post FVC, L |  | 3.80 (3.70, 3.90) | 3.64 (3.44, 3.83) | F(1, 523) = 2.02 | 0.16 |  |  |
| post FEV1/FVC, % |  | 79.46 (78.73, 80.19) | 76.14 (74.70, 77.57) | F(1, 523) = 16.34 | <0.001 |  |  |
| post PEF, L/s |  | 7.83 (7.62, 8.03) | 7.23 (6.83, 7.63) | F(1, 538) = 6.80 | <0.01 |  |  |
| post FEF25%-75%, L/s |  | 3.06 (2.94, 3.17) | 2.55 (2.32, 2.78) | F(1, 538) = 15.16 | <0.001 |  |  |
| FEV1, ppv† |  | 90.34 (88.79, 91.88) | 84.20 (81.16, 87.24) | F(1, 538) = 12.46 | <0.001 |  |  |
| FVC, ppv† |  | 93.90 (92.44, 95.35) | 91.70 (88.81, 94.60) | F(1, 523) = 1.76 | 0.19 |  |  |
| FEV1/FVC, ppv† |  | 96.66 (95.81, 97.50) | 91.03 (89.35, 92.70) | F(1, 523) = 34.55 | <0.001 |  |  |
| PEF, ppv† |  | 97.24 (95.50, 98.98) | 92.44 (89.02, 95.85) | F(1, 538) = 6.03 | 0.02 |  |  |
| FEF25%-75%, ppv† |  | 84.55 (81.90, 87.20) | 71.28 (66.07, 76.49) | F(1, 538) = 19.79 | <0.001 |  |  |
|  |  |  |  |  |  |  |  |
| ***Independent variables*** |  |  |  |  |  |  |  |
| Intra-abdominal fat, % | No test | 39.58 (37.26, 41.90) | 39.44 (34.92, 43.96) | F(5,692) = 1.16 | 0.33 | F(2,692) = 1.71 | 0.18 |
|  | Unacceptable | 38.81 (35.92, 41.70) | 33.06 (27.66, 38.47) |  |  |  |  |
|  | Acceptable | 39.55 (38.57, 40.52) | 39.91 (37.99, 41.83) |  |  |  |  |
| ***Mediator variables*** |  |  |  |  |  |  |  |
| HOMA2-IR | No test | 1.35 (1.09, 1.66) | 2.01 (1.52, 2.52) | F(5,703) = 1.51 | 0.19 | F(2,703) = 3.18 | 0.04 |
|  | Unacceptable | 1.39 (1.06, 1.71) | 1.71 (1.09, 2.34) |  |  |  |  |
|  | Acceptable | 1.40 (1.30, 1.59) | 1.32 (1.11, 1.54) |  |  |  |  |
|  |  |  |  |  |  |  |  |
| SDB symptom score, range 0-12 | No test | 1.18 (0.63, 1.73) | 1.87 (0.75, 2.98) | F(5,594) = 1.74 | 0.12 | F(2,594) = 0.07 | 0.93 |
|  | Unacceptable | 1.54 (0.83, 2.25) | 2.09 (0.79, 3.40) |  |  |  |  |
|  | Acceptable | 0.96 (0.74, 1.18) | 1.39 (0.93, 1.85) |  |  |  |  |
| ***Covariates*** |  |  |  |  |  |  |  |
| Female, % | No test | 0.36 (0.26, 0.48) | 0.76 (0.54, 0.90) | LR Chi2(5) = 22.43 | <0.001 | LR Chi2(2) = 7.62 | 0.02 |
|  | Unacceptable | 0.55 (0.41, 0.68) | 0.47 (0.24, 0.77) |  |  |  |  |
|  | Acceptable | 0.58 (0.54, 0.63) | 0.67 (0.58, 0.75) |  |  |  |  |
|  |  |  |  |  |  |  |  |
| Age, years | No test | 53.68 (50.11, 57.25) | 50.71 (43.87, 57.54) | F(5,720) = 3.70 | <0.01 | F(2,720) = 4.55 | 0.01 |
|  | Unacceptable | 53.42 (49.04, 57.80) | 36.68 (28.60, 44.76) |  |  |  |  |
|  | Acceptable | 49.85 (48.37, 51.33) | 48.13 (45.21, 51.46) |  |  |  |  |
|  |  |  |  |  |  |  |  |
| Respiratory medication | No test | 0.03 (0.01, 0.10) | 0.38 (0.20, 0.60) | LR Chi2(5) = 81.80 | <0.001 | LR Chi2(2) = 0.73 | 0.70 |
| past 24 hours, % | Unacceptable | 0.02 (0.00, 0.13) | 0.27 (0.10, 0.53) |  |  |  |  |
|  | Acceptable | 0.03 (0.02, 0.05) | 0.27 (0.20, 0.36) |  |  |  |  |
|  |  |  |  |  |  |  |  |
| Current smoker, % | No test | 0.30 (0.21, 0.50) | 0.24 (0.10, 0.46) | LR Chi2(5) = 2.29 | 0.81 | LR Chi2(2) = 0.56 | 0.75 |
|  | Unacceptable | 0.22 (0.12, 0.35) | 0.27 (0.10, 0.53) |  |  |  |  |
|  | Acceptable | 0.22 (0.19, 0.26) | 0.24 (0.17, 0.33) |  |  |  |  |
|  |  |  |  |  |  |  |  |
| Past smoker, % | No test | 0.30 (0.21, 0.50) | 0.24 (0.10, 0.46) | LR Chi2(5) = 2.35 | 0.80 | LR Chi2(2) = 1.07 | 0.59 |
|  | Unacceptable | 0.33 (0.22, 0.47) | 0.27 (0.10, 0.53) |  |  |  |  |
|  | Acceptable | 0.33 (0.29, 0.38) | 0.37 (0.29, 0.46) |  |  |  |  |
|  |  |  |  |  |  |  |  |
| Never smoker, % | No test | 0.40 (0.30, 0.52) | 0.52 (0.32, 0.72) | LR Chi2(5) = 2.35 | 0.80 | LR Chi2(2) = 1.92 | 0.38 |
|  | Unacceptable | 0.45 (0.32, 0.59) | 0.47 (0.24, 0.77) |  |  |  |  |
|  | Acceptable | 0.45 (0.40, 0.49) | 0.39 (0.30, 0.48) |  |  |  |  |
|  |  |  |  |  |  |  |  |
| Height, cm | No test | 170.17 (168.13, 172.22) | 163.47 (159.55, 167.38) | F(5,716) = 3.02 | 0.01 | F(2,716) = 3.82 | 0.02 |
|  | Unacceptable | 169.64 (167.13, 172.15) | 171.95 (167.32, 176.58) |  |  |  |  |
|  | Acceptable | 167.99 (167.14, 168.84) | 167.01 (165.33, 168.69) |  |  |  |  |
|  |  |  |  |  |  |  |  |
| Gross Annual Household Income |  |  |  |  |  |  |  |
| upto 40,000 AUD | No test | 0.49 (0.38, 0.64) | 0.43 (0.24, 0.64) | LR Chi2(5) = 9.50 | 0.09 | LR Chi2(2) = 1.33 | 0.52 |
|  | Unacceptable | 0.47 (0.34, 0.66) | 0.40 (0.19, 0.65) |  |  |  |  |
|  | Acceptable | 0.34 (0.30, 0.38) | 0.39 (0.31, 0.48) |  |  |  |  |
|  |  |  |  |  |  |  |  |
| 40,001 to 80,000 AUD | No test | 0.18 (0.11, 0.28) | 0.14 (0.05, 0.36) | LR Chi2(5) = 6.13 | 0.29 | LR Chi2(2) = 0.39 | 0.82 |
|  | Unacceptable | 0.28 (0.17, 0.41) | 0.33 (0.15, 0.59) |  |  |  |  |
|  | Acceptable | 0.28 (0.24, 0.32) | 0.30 (0.22, 0.39) |  |  |  |  |
|  |  |  |  |  |  |  |  |
| >80,000 AUD | No test | 0.22 (0.14, 0.33) | 0.19 (0.07, 0.41) | LR Chi2(5) = 7.83 | 0.17 | LR Chi2(2) = 0.21 | 0.90 |
|  | Unacceptable | 0.14 (0.07, 0.26) | 0.13 (0.03, 0.45) |  |  |  |  |
|  | Acceptable | 0.27 (0.23, 0.31) | 0.20 (0.14, 0.28) |  |  |  |  |
|  |  |  |  |  |  |  |  |
| Unknown | No test | 0.10 (0.05, 0.19) | 0.24 (0.10, 0.46) | LR Chi2(5) = 2.61 | 0.76 | LR Chi2(2) = 1.90 | 0.39 |
|  | Unacceptable | 0.12 (0.05, 0.24) | 0.13 (0.034 0.45) |  |  |  |  |
|  | Acceptable | 0.11 (0.09, 0.15) | 0.11 (0.07, 0.19) |  |  |  |  |
| Number of household residents, % |  |  |  |  |  |  |  |
| Single person | No test | 0.35 (0.25, 0.46) | 0.29 (0.13, 0.58) | LR Chi2(5) = 5.58 | 0.35 | LR Chi2(2) = 1.27 | 0.53 |
|  | Unacceptable | 0.35 (0.24, 0.49) | 0.33 (0.15, 0.59) |  |  |  |  |
|  | Acceptable | 0.25 (0.22, 0.30) | 0.31 (0.24, 0.43) |  |  |  |  |
|  |  |  |  |  |  |  |  |
| Two | No test | 0.34 (0.24, 0.45) | 0.33 (0.17, 0.55) | LR Chi2(5) = 4.57 | 0.47 | LR Chi2(2) = 1.54 | 0.46 |
|  | Unacceptable | 0.41 (0.29, 0.56) | 0.20 (0.07, 0.47) |  |  |  |  |
|  | Acceptable | 0.40 (0.36, 0.45) | 0.35 (0.27, 0.44) |  |  |  |  |
|  |  |  |  |  |  |  |  |
| Three | No test | 0.17 (0.10, 0.27) | 0.10 (0.02, 0.31) | LR Chi2(5) = 4.52 | 0.48 | LR Chi2(2) = 2.35 | 0.31 |
|  | Unacceptable | 0.10 (0.04, 0.22) | 0.20 (0.07, 0.47) |  |  |  |  |
|  | Acceptable | 0.13 (0.10, 0.16) | 0.18 (0.12, 0.26) |  |  |  |  |
|  |  |  |  |  |  |  |  |
| Four | No test | 0.12 (0.06, 0.30) | 0.24 (0.10, 0.46) | LR Chi2(5) = 4.63 | 0.46 | LR Chi2(2) = 3.12 | 0.21 |
|  | Unacceptable | 0.10 (0.04, 0.22) | 0.07 (0.01, 0.35) |  |  |  |  |
|  | Acceptable | 0.14 (0.11, 0.17) | 0.10 (0.05, 0.17) |  |  |  |  |
|  |  |  |  |  |  |  |  |
| Five or more | No test | 0.03 (0.01, 0.10) | 0.05 (0.01, 0.27) | LR Chi2(5) = 7.90 | 0.16 | LR Chi2(2) = 4.40 | 0.11 |
|  | Unacceptable | 0.04 (0.01, 0.14) | 0.20 (0.07, 0.47) |  |  |  |  |
|  | Acceptable | 0.08 (0.06, 0.11) | 0.06 (0.03, 0.12) |  |  |  |  |

Supplementary Table 1. A description of the outcome, independent and mediator variables, and covariates by current doctor-diagnosed asthma and spirometry completion and quality, The Whyalla Intergenerational Study of Health (WISH, 2008-2009).

Determination of ‘unacceptable test’ was based on either baseline and post-bronchodilator EasyOne™ system quality control (QC) grade of D or lower, or non-reproducible trials exceeding 3% variability. The analytical sample satisfied a QC grade of C or higher and reproducible trials within 3% variability. †Percentage of Predicted Value (PPV) was based on equations of Gore et al. (1995)[17].
